# Park 7: A Novel Therapeutic Target for Macrophages in Sepsis-Induced Immunosuppression

**DOI:** 10.3389/fimmu.2018.02632

**Published:** 2018-11-13

**Authors:** Yanwei Cheng, Tony N. Marion, Xue Cao, Wanting Wang, Yu Cao

**Affiliations:** ^1^West China Hospital Emergency Department, State Key Laboratory of Biotherapy, West China Hospital, Sichuan University, and Collaborative Innovation Center of Biotherapy, Chengdu, China; ^2^Disaster Medicine Center, Sichuan University, Chengdu, China; ^3^Department of Rheumatology and Immunology, West China Hospital, Sichuan University, Chengdu, China; ^4^Department of Microbiology, Immunology, and Biochemistry, The University of Tennessee Health Science Center, Memphis, TN, United States

**Keywords:** Park 7, sepsis-induced immunosuppression, inflammation, macrophages, ROS, p47^**phox**^, NADPH, crystal structure

## Abstract

Sepsis remains a serious and life-threatening condition with high morbidity and mortality due to uncontrolled inflammation together with immunosuppression with few therapeutic options. Macrophages are recognized to play essential roles throughout all phases of sepsis and affect both immune homeostasis and inflammatory processes, and macrophage dysfunction is considered to be one of the major causes for sepsis-induced immunosuppression. Currently, Parkinson disease protein 7 (Park 7) is known to play an important role in regulating the production of reactive oxygen species (ROS) through interaction with p47^phox^, a subunit of NADPH oxidase. ROS are key mediators in initiating toll-like receptor (TLR) signaling pathways to activate macrophages. Emerging evidence has strongly implicated Park 7 as an antagonist for sepsis-induced immunosuppression, which suggests that Park 7 may be a novel therapeutic target for reversing immunosuppression compromised by sepsis. Here, we review the main characteristics of sepsis-induced immunosuppression caused by macrophages and provide a detailed mechanism for how Park 7 antagonizes sepsis-induced immunosuppression initiated by the macrophage inflammatory response. Finally, we further discuss the most promising approach to develop innovative drugs that target Park 7 in patients whose initial presentation is at the late stage of sepsis.

## Introduction

Sepsis is a common clinical disease with high morbidity and mortality. Annually, ~30 million (1) people are affected by sepsis and more than 6–8 million (2) of those affected die. Despite significant advances in treatment, sepsis is still a major clinical problem and remains the leading cause of death in the critically ill patient population (3, 4) with an associated severe cost burden (5). In 2013, sepsis was responsible for more than $23 billion (6) of hospital costs in the USA alone. Thus, sepsis has been described as “the quintessential medical disorder of the twenty-first century.” On 26 May 2017, the World Health Organization listed sepsis as a global health priority by adopting a resolution to improve the prevention, diagnosis and management of this deadly disease (7).

In the recent “sepsis-3” consensus (8), sepsis is defined as a life-threatening, multiorgan dysfunction caused by a dysregulated host response to infection, which is primarily caused by Gram-negative bacteria. However, a global study of 14,000 critically ill patients found that 47% of isolates were Gram-positive, indicating that more patients currently become septic from Gram-positive infections (9). Even after an inciting infection has been resolved, septic patients continue to mount an excessive inflammatory response (10) that leads to tissue damage and organ failure. Key advances have made earlier recognition and treatment of sepsis feasible with the result that some patients can restore immune homeostasis, completely clear infection, and achieve complete recovery (11). Otherwise, patients progress into late stage sepsis and suffer from severe immunosuppression characterized by an impaired activation of the immune response and a hypo-inflammatory response (12), resulting in more difficult recovery and poor long-term outcomes with risk of cognitive and physical impairments, even an increased incidence of delayed death due to the lack of effective treatment for sepsis-induced immunosuppression (13). At present, immunosuppression in septic patients constitutes an important focus of research. Thus far, various interrelated, non-mutually exclusive mechanisms have been proposed to explain sepsis-induced immunosuppression, including cellular apoptosis (14), autophagy (15, 16), regulation by the central nervous system (17, 18), metabolic reprogramming (19), epigenetic regulation (20–22), and endotoxin tolerance (23–25). The immunopathogenesis of sepsis-induced immunosuppression is a very complex process that involves both innate and adaptive immune cells. In fact, it is at least partially caused by the dysfuction of macrophages.

## Macrophages and sepsis-induced immunosuppression

Macrophages play essential roles throughout all phases of sepsis with their ubiquitous presence and comprehensive effects on immune homeostasis and inflammatory process. After infection, macrophage is activated through Toll-like receptor (TLR) that recognizes pathogen-associated molecular patterns (PAMPs) of the invading pathogen, such as lipopolysaccharide (LPS) in Gram-negative bacteria and lipoteichoic acid (LTA)/peptidoglycan (PGN) in Gram-positive bacteria (26). In the early stage of sepsis, macrophages undergo M1 differentiation and promote host defense by eliminating invading pathogens or damaged tissues and releasing massive amounts of pro-inflammatory cytokines such as tumor necrosis factor alpha (TNF-a), interleukin-1 (IL-1), interleukin-6 (IL-6) and interleukin-8 (IL-8) (27). However, macrophages may be excessively activated during the early phase and produce excessive pro-inflammatory cytokines (28), which have been identified as one of the major causes for the high mortality rate in the early stage of sepsis (29). If macrophage-mediated pro-inflammatory responses cannot be adequately regulated, a cytokine storm may emerge (30) with the pro-inflammatory response becoming pathogenic and eventually immunosuppressive in late stage sepsis (31–33). As activated pro-inflammatory macrophages undergo apoptosis and/or polarize to the M2 phenotype that dampens the pro-inflammatory response, they may contribute to immunosuppression. Due to the cytokine storm, a large number of apoptosis-inducing factors are generated and released, including TNF-a, high mobility group box-1 protein (HMGB1) (34), thereby inducing and promoting macrophage apoptosis (35). Previous studies (36, 37) have determined the presence of an excessive level of macrophages apoptosis in human autopsies and animal models of sepsis. However, escaped M1 macrophages from apoptosis convert into M2 macrophages, showing downregulated inflammatory cytokines but upregulated anti-inflammatory cytokines (38). Certain cytokines (i.e., TNF-a, IL-13, IL-4, IL-10 etc.) can stimulate the polarization of macrophages toward M2 phenotype (39–41). Porta et al. (42) found that LPS-tolerant macrophages have the same characteristics as M2 macrophages. When a gram-negative infection persists, long-term accumulation of LPS can reprogram inflammatory responses (43) from activation to suppression leading to decreased production of inflammatory cytokines (44). The affected host may present a LPS-tolerant state, and macrophages also display the phenomenon of LPS-tolerance (45–47). In addition, M2 phenotype macrophages also accelerate T cell apoptosis and suppress Th1 cell responses (48). Collectively, this “dysfunctional” macrophage plays a key role in the pathogenesis of sepsis-induced immunosuppression because their pro-inflammatory cytokine secretions to support effective immune reactivity against primary or secondary pathogens is compromised. Therefore, modulating homeostasis of pro- and anti-inflammatory responses and functional stabilities of macrophages can be of great benefits for sepsis-induced immunosuppression.

## Reactive oxygen species (ROS) and macrophages

In addition to its cytotoxic function, reactive oxygen species (ROS) can initiate multiple signal transduction cascades to modulate macrophage function and are critical to the regulation of immune responses against pathogens (49). Previous studies have shown that ROS have an established role in regulating TLR signaling pathways, such as TLR/NF-κB and TLR/MARKs pathways (50–52). In LPS-tolerant macrophages, LPS tolerance blunts the TLR4 signaling, inhibiting the activation of the NF-κB signaling pathway downstream of TLR4, resulting in reduced production of inflammatory cytokines in response to LPS challenge (53–55). ROS can modulate the production of pro-inflammatory cytokines from LPS-tolerant macrophages by activating TLR4/NF-κB and TLR/MARKs pathways (49) mainly by accelerating the phosphorylation of IκBα and MAPK phosphatases (56, 57), respectively. In addition, it has been reported that TLR2-deficient macrophages lacked the response to Gram-positive LTA and PGN (58, 59), which can interact with TLR2, leading to NF-κB activation and induction of proinflammatory mediators in macrophages (59, 60). Rajamani (61) also demonstarted that high glucose mediated ROS could induce TLR-2 activation and downstream NF-κB signaling mediating increased inflammation during diabetic retinopathy. TLR4/NF-κB pathway also plays a central role in the regulation of macrophage polarization (48). M1 macrophage polarization is related to the activation of the TLR4/NF-κB pathway (62), whereas M2 macrophage polarization is associated with the down-regulatation of NF-κB pathway (63). A recent study has confirmed that the p50 subunit of NF-κB inhibits the NF-κB pathway and M1 polarization (42). Kuchler et al. (64) reported that impaired ROS formation contributed to an M2 phenotype shift of macrophages in sepsis by inhibiting NF-κB signaling. Consequently, increased ROS formation may reduce the M2 polarization of macrophages and protect against sepsis-induced immunosuppression.

The TLR4/MARKs pathway is also involved in regulating the LPS/pro-inflammatory cytokines-induced autophagy (65). Autophagy can induce cell death but can also be a cytoprotective process. Deficient autophagy suppresses the immune response in sepsis and increases mortality (15, 16, 66). Macrophage autophagy is considered an important part of the host immune defense, eliminating intracellular pathogens through heterophagy. It has been reported that ROS can influence the MAPK pathways to activate macrophage autophagy. In hepatoma cells, migration inhibitory factor, produced by many cells including macrophages, induced autophagy via ROS generation (67). Likewise, autophagy also participates in regulating functions of macrophages and affects their ability to defend and clear pathogens through activating NF-κB pathway (68) and enhancing phagocytic capacity of macrophages (69). All of this suggests that ROS can activate macrophages to improve bactericidal and autophagy and increase production of pro-inflammatory cytokines, thereby helping to maintain immune homeostasis. Thus, a novel approach to improve ROS production in macrophages may be a useful therapy for sepsis-induced immunosuppression.

## Parkinson disease 7

Parkinson disease 7 (Park 7), also known as DJ-1 (70), is highly conserved in almost all organisms and is ubiquitously expressed in all tissues and organs (71). Park 7 was initially discovered as a novel oncogene product (72) and is considered as a major causal factor for the early onset of Parkinson's disease (73). In the past two decades, Park 7 has been intensely studied in many diseases including cancer (74), neurodegenerative disorderes (75) and stroke (76). Among these diseases, Park 7 not only serves as a reliable predictor of auxiliary diagnosis, but also is a useful therapeutic target. Park 7 is a multi-functional protein with transcriptional regulation, protein chaperone, protease, and antioxidative stress functions (77). At present, increasing evidence has demonstrated that Park 7 plays important functions in protecting neurons (78), astrocytes (79), cardiomyocytes (80, 81), and renal proximal tubule cells (82) against oxidative stress-induced cell injury. In addition, Park 7 played an important role in restoring impaired autophagy and ameliorated phenylephrine-induced cardiac hypertrophy in a repression of cardiac hypertrophy model (83). Oxidative stress is strongly related to inflammation and is thought to be involved in the processes of many diseases, including sepsis (84). Recently, accumulating lines of evidence for Park 7 in activating the inflammatory response through modulating ROS regulating oxidative stress have also been reported (53, 85). As an antioxidant, Park 7 helps to limit to cell and tissue injury in a number of diseases by removing accumulated ROS (82, 86–89). However, studies had shown that Park 7 surprisingly seems to be required for high intracellular ROS production (85, 90). Therefore, Park 7 plays a dual role in buffering cellular ROS levels: functions as a scavenger in high ROS levels, whereas helps ROS production when essential ROS are required. In view of the hypo-inflammation characteristics of sepsis-induced immunosuppression and the critical role of Park 7 in modulating ROS production and initiating an inflammatory response, recently it has been reported that Park 7 can protect against sepsis-induced immunosuppression.

## Park 7 protects against sepsis-induced immunosuppression (figure 1)

In a park 7 knock-out (KO) mouse injected with LPS, Liu et al. (53) found that park 7 KO mice present immunosuppression phenotypes similar to the late stage of sepsis but not acute inflammation state, suggesting that park7 KO mice could serve as an animal model of sepsis-induced immunosuppression. In this model, Park 7 absence led to macrophage paralysis that resulted in increased abdominal bacterial burdens, reduced local and systemic inflammation, and impaired pro-inflammatory cytokines induction, eventually leading to high susceptibility to LPS. Neutrophil paralysis, similar to macrophage paralysis described above, was described in experimental studies of patients and sepsis animal models and was associated with decreased production of ROS in neutrophils (91, 92). In a liver fibrosis model, Park 7 deficiency inhibited ROS production in macrophages (93). Similarly, Liu et al. also observed greatly reduced ROS production in macrophages from park 7 KO mice (53). Macrophages with Park 7 deficiency showed downregulation of NF-κB and MAPK signaling pathways downstream of TLR suggesting that Park 7 deficiency can reduce the ROS production to limit TLR signaling and impair the activation of macrophages. Restoration of Park 7 expression with an inducible Park7 transgene restored the production of ROS in Park 7 KO macrophages to subsequently restore TLR signaling, pro-inflammatory cytokine production, bactericidal function, and eventually improve survival of the Park 7 KO mice in the late stage of sepsis. However, immunosuppressive IL-10 was not simultaneously enhanced after restoration of Park7 expression. During the late stage of sepsis, Park 7 may also enhance the macrophage functions by restoring impaired macrophages autophay through increased ROS and TLR/MARK signaling. Macrophage autophagy can affect cell death via complex pathways involving crosstalk with apoptosis, which may also partly attenuate immunosuppression (94). Moreover, Park 7 may contribute to the M1 macrophages polarization and inhibit the M2 macrophages polarization by the increased ROS.

**Figure 1:**
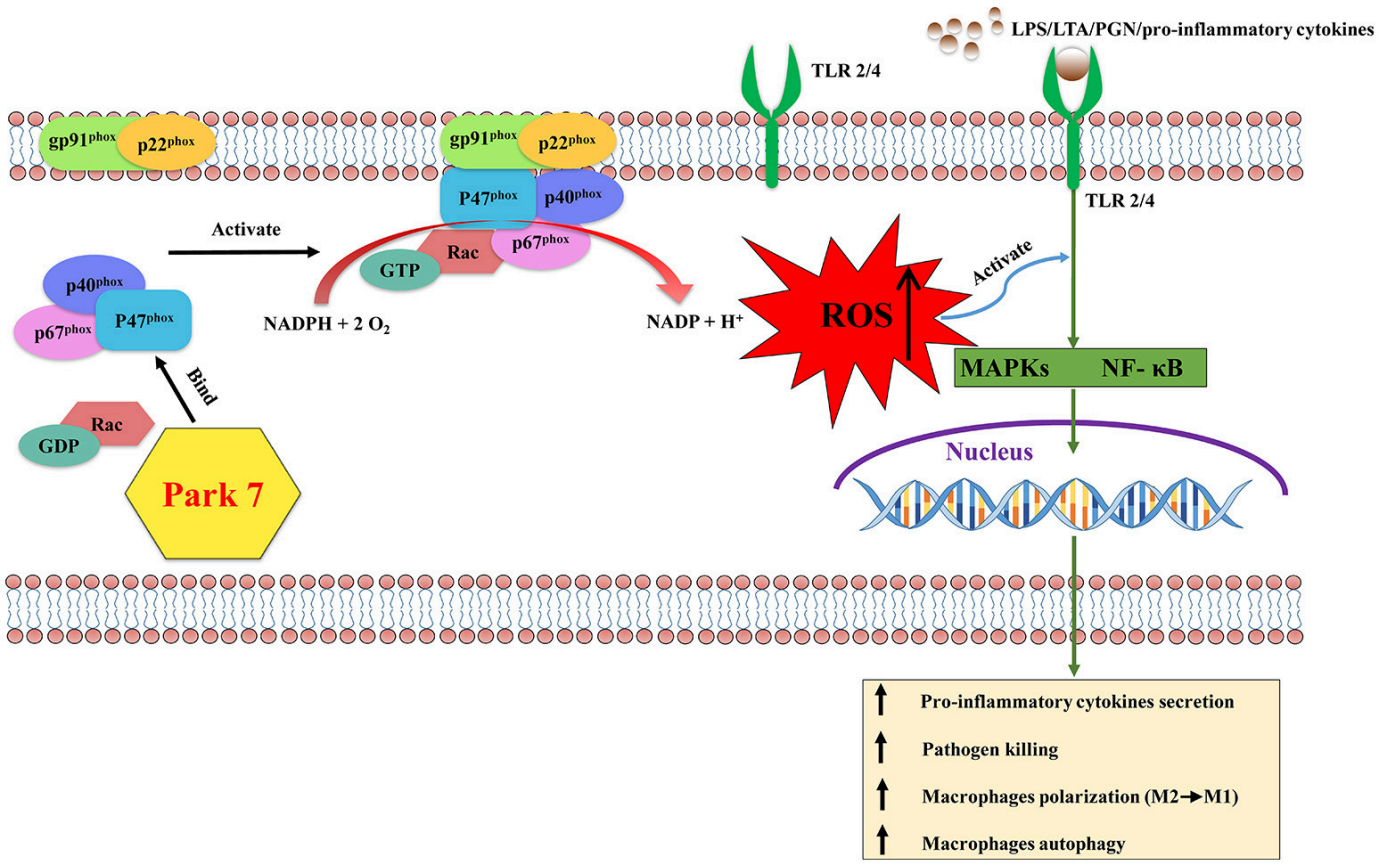
The effects of Park 7 on macrophages in sepsis-induced immunosuppression. During the late stage of sepsis, the activation of macrophages is impaired due to the blunted TLR/NF-κB and/or TLR/MARKs signaling pathways induced with LPS/LTA/PGN/pro-inflammatory cytokines. p47^phox^, a proenzyme subunit of NADPH oxidase, is key to the assembly process of NADPH oxidase. Park 7 can interact with p47^phox^ and promote its phosphorylation and membrane translocation to form the holoenzyme complex. Subsequently the activation of NADPH oxidase produces ROS, which can activate the MAPKs and NF-κB signaling pathways downstream of TLR signaling, resulting in the activation of macrophages. Activated macrophages protect against sepsis-induced immunosuppression by releasing pro-inflammatory cytokines, killing pathogen, polarizing to M1 phenotype and the enhanced capacity of autophagy. Park 7, Parkinson disease 7; LPS, lipopolysaccharide; LTA, lipoteichoic acid; PGN, peptidoglycan; ROS, reactive oxygen species; TLR, Toll-like receptor; NF-κB, nuclear factor kappa-light-chain-enhancer of activated B cells; MAPKs, mitogen-activated protein kinases.

Although there are many sources of ROS within macrophages, NADPH oxidase-derived ROS are critical in host defense. When macrophages are stimulated by an extracellular stimulus such as hormones, cytokines, and other inflammatory factors, the six proenzyme subunits of NADPH oxidase (95), including p22^phox^, gp91^phox^, GTPase Rac, p40^phox^, p47^phox^, and p67^phox^, form the holoenzyme complex that catalyzes the transfer of NADPH electrons to oxygen molecules to produce ROS (96). Key to the assembly process of the holoenzyme complex is p47^phox^ (97, 98). After macrophages are stimulated extracellularly, p47^phox^, which resides in the cytosol during the resting state (95), is phosphorylated and translocated to the plasma membrane together with the remaining proenzyme subunits and activation of NADPH oxidase (99, 100). Consistent with Liu's study (53), by interacting with p47^phox^ and modulating phosphorylation and membrane translocation of p47^phox^, Park7 promoted NADPH oxidase assembly and induced the production of ROS in macrophages. This mechanism supports the hypothesis that Park 7-targeted therapy maybe useful in the future in the treatment of sepsis-induced immunosuppression.

## Is park 7 a potential target for drug treatment in the future?

In this decade, many reports have shown the therapeutic potency of Park 7 and Park 7-targeting molecules/compounds in treating several neurodegenerative disorders (101–103). Can Park 7 be a potential target for drug treatment for sepsis-induced immunosuppression in the future? Structure-based drug design (SBDD) (104), as a valuable pharmaceutical lead discovery tool, opens up new opportunities for drug design for the patient with sepsis-induced immunosuppression. A typical example is the successful design of many valuable drugs by SBDD based on the crystal structure of Class B G-protein-coupled receptors (105). As noted above, the interaction of Park 7 and p47^phox^ is a decisive factor in activating macrophages to ameliorate sepsis-induced immunosuppression, suggesting that the interaction between Park 7 and p47^phox^ may be an ideal target for drug design. Single crystal structures of Park 7 and p47^phox^ have been determined. Human Park 7 consists of 189 amino acids from N-terminus to C-terminus, which folds into a helix-strand-helix sandwich structure (106). The C-terminal domain (CTD) of Park 7 physically interacts with p47^phox^ *in vitro* (53). In addition, the C106 and L166 residues in the CTD of Park 7 are important for its functions (107, 108), suggesting the two residues might play a key role in Park 7 interacting with p47^phox^. However, the details of the interaction depend on the crystal structure of the Park7-p47^phox^ complex. Therefore, determing Park7-p47^phox^ complex structure should be an urgent issue for future research.

With regard to a potential drug treatment based on Park 7 in the future it might be important to discuss three relevant points here. (1) It would be necessary to detect/diagnose the immune status of the patient in sepsis-induced immunosuppression. (2) In line with this it would be crucial to find the right timing to start drug treatment to overcome sepsis-induced immunosuppression. (3) Considering the complexity of the host response during sepsis and the variety of pathophysiological pathways involved, it is unlikely that the current “one-target” and “one-size-fits-all” approach will ever be successful. To date, absolute lymphocyte count and decreased expression of HLA-DR by monocytes seem to be the most robust markers for patient stratification in multicenter clinical trials (109–112). Measurement of soluble mediators such as IL-6, IL-10, and TNF-a can also help detect immune status. However, a convenient, faster detection protocol and other effective drugs are extremely necessary. These are interesting issues that are worth pursuing in the future.

## Conclusion

In summary, macrophages, as one of the most important cells of the innate immune system, play an important role in inflammatory and immune processes. In the early stage of sepsis, macrophages usually have a pro-inflammatory phenotype, whereas the excessive inflammatory macrophage response can lead to macrophages apoptosis and change macrophage polarization contributing to the immunosuppression. ROS have the capacity to initiate many TLR signaling pathways and in turn modulate macrophage functions and are produced by the activation of NADPH oxidase. Park 7 has been extensively studied in many diseases and can serve as an effective therapeutic target. For research on sepsis in the late stage, Park7 KO mice can be an ideal model. The interaction of Park7 and p47^phox^ can activate NADPH oxidase and subsequently increase ROS in macrophages to initiate TRL signaling to in turn, reinforce macrophage functions to protect against sepsis–induced immunosuppression. In light of this understanding, the Park 7/p47^phox^/ROS axis may become an effective therapeutic target for sepsis induced immunosuppression.

## Author contributions

YaC wrote the first draft of this article. XC and WW designed the figure. YC and TM critically revised the manuscript for important intellectual content. All authors approved the final version.

### Conflict of interest statement

The authors declare that the research was conducted in the absence of any commercial or financial relationships that could be construed as a potential conflict of interest.

## References

[1] Fleischmann C Scherag A Adhikari NK Hartog CS Tsaganos T Schlattmann P. Assessment of Global Incidence and Mortality of Hospital-treated Sepsis. Current estimates and limitations. Am J Respir Crit Care Med. (2016) 193:259–72. 10.1164/rccm.201504-0781OC 26414292

[2] Seymour CW Rea TD Kahn JM Walkey AJ Yealy DM Angus DC. Severe sepsis in pre-hospital emergency care: analysis of incidence, care, and outcome. Am J Respir Crit Care Med. (2012) 186:1264–71. 10.1164/rccm.201204-0713OC 23087028 PMC3622444

[3] Dombrovskiy VY Martin AA Sunderram J Paz HL. Rapid increase in hospitalization and mortality rates for severe sepsis in the United States: a trend analysis from 1993 to 2003. Crit Care Med. (2007) 35:1244–50. 10.1097/01.ccm.0000261890.41311.e9 17414736

[4] Melamed A Sorvillo FJ. The burden of sepsis-associated mortality in the United States from 1999 to 2005: an analysis of multiple-cause-of-death data. Crit Care (2009) 13:R28. 10.1186/cc7733 19250547 PMC2688146

[5] Tiru B DiNino EK Orenstein A Mailloux PT Pesaturo A Gupta A. The Economic and Humanistic burden of severe sepsis. Pharmacoeconomics (2015) 33:925–37. 10.1007/s40273-015-0282-y 25935211

[6] Torio CM Moore BJ National Inpatient Hospital Costs: The Most Expensive Conditions by Payer, 2013: Statistical Brief #204, in Healthcare Cost and Utilization Project (HCUP) Statistical Briefs. (2006). Rockville, MD: Agency for Healthcare Research and Quality (US). 27359025

[7] Reinhart K Daniels R Kissoon N Machado FR Schachter RD Finfer S. Recognizing sepsis as a global health priority - A WHO resolution. N Engl J Med. (2017) 377:414–7. 10.1056/NEJMp1707170 28658587

[8] Singer M Deutschman CS Seymour CW Shankar-Hari M Annane D Bauer M The third international consensus definitions for sepsis and septic shock (Sepsis-3). JAMA (2016) 315:801–10. 10.1001/jama.2016.0287 26903338 PMC4968574

[9] Vincent JL Rello J Marshall J Silva E Anzueto A Martin CD. International study of the prevalence and outcomes of infection in intensive care units. JAMA (2009) 302:2323–9. 10.1001/jama.2009.1754 19952319

[10] van der Poll T van de Veerdonk FL Scicluna BP Netea MG The immunopathology of sepsis and potential therapeutic targets. Nat Rev Immunol. (2017) 17:407–20. 10.1038/nri.2017.36 28436424

[11] Kumar A Kethireddy S. Emerging concepts in optimizing antimicrobial therapy of septic shock: speed is life but a hammer helps too. Crit Care (2013) 17:104. 10.1186/cc11890 23320914 PMC4056640

[12] Hotchkiss RS Monneret G Payen D. Immunosuppression in sepsis: a novel understanding of the disorder and a new therapeutic approach. Lancet Infect Dis. (2013) 13:260–8. 10.1016/s1473-3099(13)70001-x 23427891 PMC3798159

[13] Iwashyna TJ Ely EW Smith DM Langa KM. Long-term cognitive impairment and functional disability among survivors of severe sepsis. JAMA (2010) 304:1787–94. 10.1001/jama.2010.1553 20978258 PMC3345288

[14] Hotchkiss RS Swanson PE Freeman BD Tinsley KW Cobb JP Matuschak GM. Apoptotic cell death in patients with sepsis, shock, and multiple organ dysfunction. Crit Care Med. (1999) 27:1230–51. 10446814 10.1097/00003246-199907000-00002

[15] Lin CW Lo S Hsu C Hsieh CH Chang YF Hou BS. T-cell autophagy deficiency increases mortality and suppresses immune responses after sepsis. PLoS ONE (2014) 9:e102066. 10.1371/journal.pone.0102066 25029098 PMC4100769

[16] Oami T Watanabe E Hatano M Sunahara S Fujimura L Sakamoto A. Suppression of T cell autophagy results in decreased viability and function of T cells through accelerated apoptosis in a murine sepsis model. Crit Care Med. (2017) 45:e77–85. 10.1097/ccm.0000000000002016 27618275 PMC5364514

[17] Singer BH Newstead MW Zeng X Cooke CL Thompson RC Singer K. Cecal ligation and puncture results in long-term central nervous system myeloid inflammation. PLoS ONE (2016) 11:e0149136. 10.1371/journal.pone.0149136 26862765 PMC4749127

[18] Martelli D Yao ST McKinley MJ McAllen RM Reflex control of inflammation by sympathetic nerves, not the vagus. J Physiol. (2014) 592:1677–86. 10.1113/jphysiol.2013.268573 24421357 PMC3979618

[19] Arts RJ Gresnigt MS Joosten LA Netea MG. Cellular metabolism of myeloid cells in sepsis. J Leukoc Biol. (2017) 101:151–164. 10.1189/jlb.4MR0216-066R 27272310

[20] Cazalis MA Lepape A Venet F Frager F Mougin B Vallin H. Early and dynamic changes in gene expression in septic shock patients: a genome-wide approach. Intensive Care Med Exp. (2014) 2:20. 10.1186/s40635-014-0020-3 26215705 PMC4512996

[21] Xiao W Mindrinos MN Seok J Cuschieri J Cuenca AG Gao H. A genomic storm in critically injured humans. J Exp Med. (2011) 208:2581–90. 10.1084/jem.20111354 22110166 PMC3244029

[22] Davenport EE Burnham KL Radhakrishnan J Humburg P Hutton P Mills TC. Genomic landscape of the individual host response and outcomes in sepsis: a prospective cohort study. Lancet Respir Med. (2016) 4:259–71. 10.1016/s2213-2600(16)00046-1 26917434 PMC4820667

[23] Allantaz-Frager F Turrel-Davin F Venet F Monnin C De Saint Jean A Barbalat V Identification of biomarkers of response to IFNg during endotoxin tolerance: application to septic shock. PLoS ONE (2013) 8:e68218 10.1371/journal.pone.0068218 23874546 PMC3708924

[24] Giza DE Vasilescu C. [MicroRNA's role in sepsis and endotoxin tolerance. More players on the stage]. Chirurgia (2010) 105:625–30. 10.3109/19401736.2015.1060434 21141085

[25] Andrades ME Morina A Spasic S Spasojevic I. Bench-to-bedside review: sepsis - from the redox point of view. Crit Care (2011) 15:230. 10.1186/cc10334 21996422 PMC3334726

[26] Kumar H Kawai T Akira S. Pathogen recognition by the innate immune system. Int Rev Immunol (2011) 30:16–34. 10.3109/08830185.2010.529976 21235323

[27] Murray PJ Wynn TA Protective and pathogenic functions of macrophage subsets. Nat Rev Immunol. (2011) 11:723–37. 10.1038/nri3073 21997792 PMC3422549

[28] Wang TS Deng JC. Molecular and cellular aspects of sepsis-induced immunosuppression. J Mol Med. (2008) 86:495–506. 10.1007/s00109-007-0300-4 18259721

[29] Angus DC van der Poll T Severe sepsis and septic shock. N Engl J Med. (2013) 369:840–51. 10.1056/NEJMra1208623 23984731

[30] Rittirsch D Flierl MA Ward PA. Harmful molecular mechanisms in sepsis. Nat Rev Immunol. (2008) 8:776–87. 10.1038/nri2402 18802444 PMC2786961

[31] Nathan C Ding A. Nonresolving inflammation. Cell (2010) 140:871–82. 10.1016/j.cell.2010.02.029 20303877

[32] Sindrilaru A Peters T Wieschalka S Baican C Baican A Peter H. An unrestrained proinflammatory M1 macrophage population induced by iron impairs wound healing in humans and mice. J Clin Invest. (2011) 121:985–97. 10.1172/jci44490 21317534 PMC3049372

[33] Krausgruber T Blazek K Smallie T Alzabin S Lockstone H Sahgal N. IRF5 promotes inflammatory macrophage polarization and TH1-TH17 responses. Nat Immunol. (2011) 12:231–8. 10.1038/ni.1990 21240265

[34] Zhu XM Yao YM Liang HP Liu F Dong N Yu Y. Effect of high mobility group box-1 protein on apoptosis of peritoneal macrophages. Arch Biochem Biophys. (2009) 492:54–61. 10.1016/j.abb.2009.09.016 19800306

[35] Luan YY Yao YM Xiao XZ Sheng ZY. Insights into the apoptotic death of immune cells in sepsis. J Interferon Cytokine Res. (2015) 35:17–22. 10.1089/jir.2014.0069 25007137 PMC4291200

[36] Rimmele T Payen D Cantaluppi V Marshall J Gomez H Gomez A. Immune cell phenotype and function in sepsis. Shock (2016) 45:282–91. 10.1097/shk.0000000000000495 26529661 PMC4752878

[37] Luan YY Dong N Xie M Xiao XZ Yao YM. The significance and regulatory mechanisms of innate immune cells in the development of sepsis. J Interferon Cytokine Res. (2014) 34:2–15. 10.1089/jir.2013.0042 24006870 PMC3887438

[38] Mantovani A Sica A Locati M. Macrophage polarization comes of age. Immunity (2005) 23:344–6. 10.1016/j.immuni.2005.10.001 16226499

[39] Gordon S Martinez FO. Alternative activation of macrophages: mechanism and functions. Immunity (2010) 32:593–604. 10.1016/j.immuni.2010.05.007 20510870

[40] Sica A Mantovani A. Macrophage plasticity and polarization: in vivo veritas. J Clin Invest. (2012) 122:787–95. 10.1172/jci59643 22378047 PMC3287223

[41] Ip WKE Hoshi N Shouval DS Snapper S Medzhitov R. Anti-inflammatory effect of IL-10 mediated by metabolic reprogramming of macrophages. Science (2017) 356:513–9. 10.1126/science.aal3535 28473584 PMC6260791

[42] Porta C Rimoldi M Raes G Brys L Ghezzi P Di Liberto D. Tolerance and M2 (alternative) macrophage polarization are related processes orchestrated by p50 nuclear factor kappaB. Proc Natl Acad Sci USA. (2009) 106:14978–83. 10.1073/pnas.0809784106 19706447 PMC2736429

[43] Seeley JJ Ghosh S. Molecular mechanisms of innate memory and tolerance to LPS. J Leukoc Biol. (2017) 101:107–119. 10.1189/jlb.3MR0316-118RR 27780875

[44] Biswas SK Lopez-Collazo E. Endotoxin tolerance: new mechanisms, molecules and clinical significance. Trends Immunol. (2009) 30:475–87. 10.1016/j.it.2009.07.009 19781994

[45] Sato S Takeuchi O Fujita T Tomizawa H Takeda K Akira S. A variety of microbial components induce tolerance to lipopolysaccharide by differentially affecting MyD88-dependent and -independent pathways. Int Immunol. (2002) 14:783–91. 10.1093/intimm/dxf046 12096038

[46] Medvedev AE Kopydlowski KM Vogel SN. Inhibition of lipopolysaccharide-induced signal transduction in endotoxin-tolerized mouse macrophages: dysregulation of cytokine, chemokine, and toll-like receptor 2 and 4 gene expression. J Immunol. (2000) 164:5564–74. 10.4049/jimmunol.164.11.5564 10820230

[47] Dobrovolskaia MA Medvedev AE Thomas KE Cuesta N Toshchakov V Ren T. Induction of in vitro reprogramming by Toll-like receptor (TLR)2 and TLR4 agonists in murine macrophages: effects of TLR “homotolerance” versus “heterotolerance” on NF-kappa B signaling pathway components. J Immunol. (2003) 170:508–19. 10.4049/jimmunol.170.1.508 12496438

[48] Liu YC Zou XB Chai YF Yao YM. Macrophage polarization in inflammatory diseases. Int J Biol Sci. (2014) 10:520–9. 10.7150/ijbs.8879 24910531 PMC4046879

[49] West AP Brodsky IE Rahner C Woo DK Erdjument-Bromage H Tempst P. TLR signalling augments macrophage bactericidal activity through mitochondrial ROS. Nature (2011) 472:476–80. 10.1038/nature09973 21525932 PMC3460538

[50] Lee CC Avalos AM Ploegh HL. Accessory molecules for Toll-like receptors and their function. Nat Rev Immunol. (2012) 12:168–79. 10.1038/nri3151 22301850 PMC3677579

[51] Gallego C Golenbock D Gomez MA Saravia NG. Toll-like receptors participate in macrophage activation and intracellular control of Leishmania (Viannia) panamensis. Infect Immun. (2011) 79:2871–9. 10.1128/iai.01388-10 21518783 PMC3191987

[52] Kamata H Honda S Maeda S Chang L Hirata H Karin M. Reactive oxygen species promote TNFalpha-induced death and sustained JNK activation by inhibiting MAP kinase phosphatases. Cell (2005) 120:649–61. 10.1016/j.cell.2004.12.041 15766528

[53] Liu W Wu H Chen L Wen Y Kong X Gao WQ. Park7 interacts with p47(phox) to direct NADPH oxidase-dependent ROS production and protect against sepsis. Cell Res. (2015) 25:691–706. 10.1038/cr.2015.63 26021615 PMC4456629

[54] Hoogendijk AJ Garcia-Laorden MI van Vught LA Wiewel MA Belkasim-Bohoudi H Duitman J. Sepsis patients display a reduced capacity to activate nuclear factor-kappaB in multiple cell types. Crit Care Med. (2017) 45:e524–31. 10.1097/ccm.0000000000002294 28240686

[55] Nomura F Akashi S Sakao Y Sato S Kawai T Matsumoto M. Cutting edge: endotoxin tolerance in mouse peritoneal macrophages correlates with down-regulation of surface toll-like receptor 4 expression. J Immunol. (2000) 164:3476–9. 10.4049/jimmunol.164.7.3476 10725699

[56] Zhang J Wang X Vikash V Ye Q Wu D Liu Y. ROS and ROS-Mediated Cellular Signaling. Oxid Med Cell Longev. (2016) 2016:4350965. 10.1155/2016/4350965 26998193 PMC4779832

[57] Park J Min JS Kim B Chae UB Yun JW Choi MS. Mitochondrial ROS govern the LPS-induced pro-inflammatory response in microglia cells by regulating MAPK and NF-kappaB pathways. Neurosci Lett. (2015) 584:191–6. 10.1016/j.neulet.2014.10.016 25459294

[58] Park OJ Han JY Baik JE Jeon JH Kang SS Yun CH. Lipoteichoic acid of *Enterococcus faecalis* induces the expression of chemokines via TLR2 and PAFR signaling pathways. J Leukoc Biol. (2013) 94:1275–84. 10.1189/jlb.1012522 23964117

[59] Hong SW Baik JE Kang SS Yun CH Seo DG Han SH. Lipoteichoic acid of *Streptococcus mutans* interacts with Toll-like receptor 2 through the lipid moiety for induction of inflammatory mediators in murine macrophages. Mol Immunol. (2014) 57:284–91. 10.1016/j.molimm.2013.10.004 24216318

[60] Paul-Clark MJ McMaster SK Belcher E Sorrentino R Anandarajah J Fleet M. Differential effects of Gram-positive versus Gram-negative bacteria on NOSII and TNFalpha in macrophages: role of TLRs in synergy between the two. Br J Pharmacol. (2006) 148:1067–75. 10.1038/sj.bjp.0706815 16783405 PMC1752017

[61] Rajamani U Jialal I. Hyperglycemia induces Toll-like receptor-2 and−4 expression and activity in human microvascular retinal endothelial cells: implications for diabetic retinopathy. J Diabetes Res. (2014) 2014:790902. 10.1155/2014/790902 25610879 PMC4293793

[62] Odegaard JI Chawla A. Mechanisms of macrophage activation in obesity-induced insulin resistance. Nat Clin Pract Endocrinol Metab. (2008) 4:619–26. 10.1038/ncpendmet0976 18838972 PMC3381907

[63] Pollard JW Trophic macrophages in development and disease. Nat Rev Immunol. (2009) 9:259–70. 10.1038/nri2528 19282852 PMC3648866

[64] Kuchler L Giegerich AK Sha LK Knape T Wong MS Schroder K. SYNCRIP-dependent Nox2 mRNA destabilization impairs ROS formation in M2-polarized macrophages. Antioxid Redox Signal. (2014) 21:2483–97. 10.1089/ars.2013.5760 24844655

[65] Xu Y Jagannath C Liu XD Sharafkhaneh A Kolodziejska KE Eissa NT. Toll-like receptor 4 is a sensor for autophagy associated with innate immunity. Immunity (2007) 27:135–44. 10.1016/j.immuni.2007.05.022 17658277 PMC2680670

[66] Nakahira K Haspel JA Rathinam VA Lee SJ Dolinay T Lam HC. Autophagy proteins regulate innate immune responses by inhibiting the release of mitochondrial DNA mediated by the NALP3 inflammasome. Nat Immunol. (2011) 12:222–30. 10.1038/ni.1980 21151103 PMC3079381

[67] Chuang YC Su WH Lei HY Lin YS Liu HS Chang CP. Macrophage migration inhibitory factor induces autophagy via reactive oxygen species generation. PLoS ONE (2012) 7:e37613. 10.1371/journal.pone.0037613 22629429 PMC3358253

[68] Ren C Zhang H Wu TT Yao YM. Autophagy: a potential therapeutic target for reversing sepsis-induced immunosuppression. Front Immunol. (2017) 8:1832. 10.3389/fimmu.2017.01832 29326712 PMC5741675

[69] Lee S Lee SJ Coronata AA Fredenburgh LE Chung SW Perrella MA. Carbon monoxide confers protection in sepsis by enhancing beclin 1-dependent autophagy and phagocytosis. Antioxid Redox Signal. (2014) 20:432–42. 10.1089/ars.2013.5368 23971531 PMC3894711

[70] Bandyopadhyay S Cookson MR. Evolutionary and functional relationships within the DJ1 superfamily. BMC Evol Biol. (2004) 4:6. 10.1186/1471-2148-4-6 15070401 PMC385224

[71] Shen ZY Sun Q Xia ZY Meng QT Lei SQ Zhao B. Overexpression of DJ-1 reduces oxidative stress and attenuates hypoxia/reoxygenation injury in NRK-52E cells exposed to high glucose. Int J Mol Med. (2016) 38:729–36. 10.3892/ijmm.2016.2680 27430285 PMC4990284

[72] Nagakubo D Taira T Kitaura H Ikeda M Tamai K SM Iguchi-Ariga. DJ-1, a novel oncogene which transforms mouse NIH3T3 cells in cooperation with ras. Biochem Biophys Res Commun. (1997) 231:509–13. 10.1006/bbrc.1997.6132 9070310

[73] Bonifati V Rizzu P van Baren MJ Schaap O Breedveld GJ Krieger E. Mutations in the DJ-1 gene associated with autosomal recessive early-onset parkinsonism. Science (2003) 299:256–9. 10.1126/science.1077209 12446870

[74] Clements CM McNally RS Conti BJ Mak TW Ting JP. DJ-1, a cancer- and Parkinson's disease-associated protein, stabilizes the antioxidant transcriptional master regulator Nrf2. Proc Natl Acad Sci USA. (2006) 103:15091–6. 10.1073/pnas.0607260103 17015834 PMC1586179

[75] Honbou K Suzuki NN Horiuchi M Niki T Taira T Ariga H. The crystal structure of DJ-1, a protein related to male fertility and Parkinson's disease. J Biol Chem. (2003) 278:31380–4. 10.1074/jbc.M305878200 12796482

[76] Dongworth RK Mukherjee UA Hall AR Astin R Ong SB Yao Z. DJ-1 protects against cell death following acute cardiac ischemia-reperfusion injury. Cell Death Dis. (2014) 5:e1082. 10.1038/cddis.2014.41 24577080 PMC3944257

[77] Hijioka M Inden M Yanagisawa D Kitamura Y. DJ-1/PARK7: a new therapeutic target for Neurodegenerative disorders. Biol Pharm Bull. (2017) 40:548–52. 10.1248/bpb.b16-01006 28458339

[78] Kim W Kim DW Jeong HJ Yoo DY Jung HY Nam SM. Tat-DJ-1 protects neurons from ischemic damage in the ventral horn of rabbit spinal cord via increasing antioxidant levels. Neurochem Res. (2014) 39:187–93. 10.1007/s11064-013-1205-y 24293249

[79] Yanagida T Tsushima J Kitamura Y Yanagisawa D Takata K Shibaike T. Oxidative stress induction of DJ-1 protein in reactive astrocytes scavenges free radicals and reduces cell injury. Oxid Med Cell Longev. (2009) 2:36–42. 10.4161/oxim.2.1.7985 20046643 PMC2763229

[80] Yu HH Xu Q Chen HP Wang S Huang XS Huang QR. Stable overexpression of DJ-1 protects H9c2 cells against oxidative stress under a hypoxia condition. Cell Biochem Funct. (2013) 31:643–51. 10.1002/cbf.2949 23281015

[81] Billia F Hauck L Grothe D Konecny F Rao V Kim RH. Parkinson-susceptibility gene DJ-1/PARK7 protects the murine heart from oxidative damage in vivo. Proc Natl Acad Sci USA. (2013) 110:6085–90. 10.1073/pnas.1303444110 23530187 PMC3625258

[82] Cuevas S Zhang Y Yang Y Escano C Asico L Jones JE. Role of renal DJ-1 in the pathogenesis of hypertension associated with increased reactive oxygen species production. Hypertension (2012) 59:446–52. 10.1161/hypertensionaha.111.185744 22215708 PMC3395064

[83] Xue R Jiang J Dong B Tan W Sun Y Zhao J. DJ-1 activates autophagy in the repression of cardiac hypertrophy. Arch Biochem Biophys. (2017) 633: 124–32. 10.1016/j.abb.2017.09.012 28941803

[84] Galley HF. Oxidative stress and mitochondrial dysfunction in sepsis. Br J Anaesth. (2011) 107:57–64. 10.1093/bja/aer093 21596843

[85] Chen L Luo M Sun X Qin J Yu C Wen Y. DJ-1 deficiency attenuates expansion of liver progenitor cells through modulating the inflammatory and fibrogenic niches. Cell Death Dis. (2016) 7:e2257. 10.1038/cddis.2016.161 27277679 PMC5143389

[86] Xu X Martin F Friedman JS. The familial Parkinson's disease gene DJ-1 (PARK7) is expressed in red cells and plays a role in protection against oxidative damage. Blood Cells Mol Dis. (2010) 45:227–32. 10.1016/j.bcmd.2010.07.014 20800516 PMC2962440

[87] Zhang XL Yuan YH Shao QH Wang ZZ Zhu CG Shi JG. DJ-1 regulating PI3K-Nrf2 signaling plays a significant role in bibenzyl compound 20C-mediated neuroprotection against rotenone-induced oxidative insult. Toxicol Lett. (2017) 271:74–83. 10.1016/j.toxlet.2017.02.022 28245986

[88] Taira T Saito Y Niki T SM Iguchi-Ariga Takahashi K Ariga H. DJ-1 has a role in antioxidative stress to prevent cell death. EMBO Rep. (2004) 5:213–8. 10.1038/sj.embor.7400074 14749723 PMC1298985

[89] Amatullah H Shan Y Beauchamp BL Gali PL Gupta S T Maron-Gutierrez. DJ-1/PARK7 impairs bacterial clearance in sepsis. Am J Respir Crit Care Med. (2017) 195:889–905. 10.1164/rccm.201604-0730OC 27735193

[90] Vasseur S Afzal S Tomasini R Guillaumond F Tardivel-Lacombe J Mak TW. Consequences of DJ-1 upregulation following p53 loss and cell transformation. Oncogene (2012) 31:664–70. 10.1038/onc.2011.268 21725356

[91] Alves-Filho JC Spiller F Cunha FQ. Neutrophil paralysis in sepsis. Shock (2010) 34(Suppl. 1):15–21. 10.1097/SHK.0b013e3181e7e61b 20714263

[92] Kovach MA Standiford TJ. The function of neutrophils in sepsis. Curr Opin Infect Dis. (2012) 25:321–7. 10.1097/QCO.0b013e3283528c9b 22421753

[93] Yu Y Sun X Gu J Yu C Wen Y Gao Y. Deficiency of DJ-1 Ameliorates liver Fibrosis through inhibition of Hepatic ROS production and inflammation. Int J Biol Sci. (2016) 12:1225–35. 10.7150/ijbs.15154 27766037 PMC5069444

[94] Qiu P Liu Y Zhang J. Review: the role and mechanisms of macrophage autophagy in sepsis. Inflammation (2018) 1–14. 10.1007/s10753-018-0890-8 30194660

[95] Groemping Y Lapouge K Smerdon SJ Rittinger K. Molecular basis of phosphorylation-induced activation of the NADPH oxidase. Cell (2003) 113:343–55. 10.1016/S0092-8674(03)00314-3 12732142

[96] Babior BM. NADPH oxidase: an update. Blood (1999) 93:1464–76. 10029572

[97] de Mendez I Homayounpour N Leto TL Specificity of p47phox SH3 domain interactions in NADPH oxidase assembly and activation. Mol Cell Biol. (1997) 17:2177–85. 9121467 10.1128/mcb.17.4.2177 PMC232066

[98] Lambeth JD. NOX enzymes and the biology of reactive oxygen. Nat Rev Immunol. (2004) 4:181–9. 10.1038/nri1312 15039755

[99] El-Benna J Dang PM Gougerot-Pocidalo MA Marie JC Braut-Boucher F. p47phox, the phagocyte NADPH oxidase/NOX2 organizer: structure, phosphorylation and implication in diseases. Exp Mol Med. (2009) 41:217–25. 10.3858/emm.2009.41.4.058 19372727 PMC2679237

[100] Gao XP Standiford TJ Rahman A Newstead M Holland SM Dinauer MC. Role of NADPH oxidase in the mechanism of lung neutrophil sequestration and microvessel injury induced by Gram-negative sepsis: studies in p47phox^−/−^ and gp91phox^−/−^ mice. J Immunol. (2002) 168:3974–82. 10.4049/jimmunol.168.8.3974 11937554

[101] Kitamura Y Watanabe S Taguchi M Takagi K Kawata T Takahashi-Niki K. Neuroprotective effect of a new DJ-1-binding compound against neurodegeneration in Parkinson's disease and stroke model rats. Mol Neurodegener. (2011) 6:48. 10.1186/1750-1326-6-48 21740546 PMC3141555

[102] Lev N Barhum Y Ben-Zur T Aharony I Trifonov L Regev N. A DJ-1 based Peptide attenuates dopaminergic degeneration in mice models of Parkinson's disease via enhancing Nrf2. PLoS ONE (2015) 10:e0127549. 10.1371/journal.pone.0127549 26024237 PMC4449207

[103] Kitamura Y Inden M Kimoto Y Takata K Yanagisawa D Hijioka M. Effects of a DJ-1-binding compound on spatial learning and memory impairment in a mouse model of Alzheimer's Disease. J Alzheimers Dis. (2017) 55:67–72. 10.3233/jad-160574 27662308

[104] Grey JL Thompson DH. Challenges and opportunities for new protein crystallization strategies in structure-based drug design. Expert Opin Drug Discov. (2010) 5:1039–45. 10.1517/17460441.2010.515583 21116481 PMC2992350

[105] Zhang H Qiao A Yang D Yang L Dai A de Graaf C. Structure of the full-length glucagon class B G-protein-coupled receptor. Nature (2017) 546:259–64. 10.1038/nature22363 28514451 PMC5492955

[106] Wilson MA Collins JL Hod Y Ringe D Petsko GA. The 1.1-A resolution crystal structure of DJ-1, the protein mutated in autosomal recessive early onset Parkinson's disease. Proc Natl Acad Sci USA. (2003) 100:9256–61. 10.1073/pnas.1133288100 12855764 PMC170905

[107] Olzmann JA Brown K Wilkinson KD Rees HD Huai Q Ke H. Familial Parkinson's disease-associated L166P mutation disrupts DJ-1 protein folding and function. J Biol Chem. (2004) 279:8506–15. 10.1074/jbc.M311017200 14665635

[108] Wilson MA. The role of cysteine oxidation in DJ-1 function and dysfunction. Antioxid Redox Signal. (2011) 15:111–22. 10.1089/ars.2010.3481 20812780 PMC3110098

[109] Schefold JC. Measurement of monocytic HLA-DR (mHLA-DR) expression in patients with severe sepsis and septic shock: assessment of immune organ failure. Intensive Care Med. (2010) 36:1810–2. 10.1007/s00134-010-1965-7 20652681

[110] Cheron A Monneret G Landelle C Floccard B Allaouchiche B. [Low monocytic HLA-DR expression and risk of secondary infection]. Ann Fr Anesth Reanim. (2010) 29:368–76. 10.1016/j.annfar.2010.02.015 20356708

[111] Monneret G Venet F. Sepsis-induced immune alterations monitoring by flow cytometry as a promising tool for individualized therapy. Cytometry B Clin Cytom. (2016) 90:376–86. 10.1002/cyto.b.21270 26130241

[112] Monneret G Venet F Pachot A Lepape A. Monitoring immune dysfunctions in the septic patient: a new skin for the old ceremony. Mol Med. (2008) 14:64–78. 10.2119/2007-00102.Monneret 18026569 PMC2078557

